# Optimizing Analgesia After Minimally Invasive Cardiac Surgery: A Randomized Non-Inferiority Trial Comparing Interpectoral Plane Block Plus Serratus Anterior Plane Block to Erector Spinae Plane Block

**DOI:** 10.3390/jcm14113786

**Published:** 2025-05-28

**Authors:** Onur Baran, Ayhan Şahin, Selami Gürkan, Özcan Gür, Cavidan Arar

**Affiliations:** 1 Department of Anesthesiology and Reanimation, Faculty of Medicine, Tekirdağ Namık Kemal University, Tekirdağ 59030, Türkiye; aysahin@nku.edu.tr (A.Ş.); mcarar@nku.edu.tr (C.A.); 2 Department of Cardiovascular Surgery, Faculty of Medicine, Tekirdağ Namık Kemal University, Tekirdağ 59030, Türkiye; sgurkan@nku.edu.tr (S.G.); ozcangur@hotmail.com (Ö.G.)

**Keywords:** analgesia, erector spinae plane block, interpectoral plane block, minimally invasive cardiac surgery, pectoserratus plane block, superficial serratus anterior plane block

## Abstract

**Background:** Regional anesthesia techniques are increasingly used for pain management in minimally invasive cardiac surgery (MICS). We aimed to evaluate whether the combination of interpectoral plane block (IPB) and superficial serratus anterior plane block (SAPB) provides non-inferior postoperative analgesia compared to erector spinae plane block (ESPB) in adult patients undergoing MICS. **Methods:** In this prospective, single-center, double-blind, randomized, non-inferiority trial, 40 adult patients scheduled for MICS were allocated to receive either ESPB (*n* = 20) or a combination of IPB + SAPB (*n* = 20) prior to surgical incision. All patients received standardized anesthesia. Pain was assessed using the Critical-Care Pain Observation Tool (CPOT) during intubation and the Numerical Rating Scale (NRS) at 6–48 h postoperatively, following extubation. The primary outcome was the NRS score at 24 h. A non-inferiority margin of 2 NRS points was pre-specified, and non-inferiority was evaluated using between-group differences with 95% confidence intervals. Opioid consumption was recorded via PCA fentanyl and rescue analgesics, converted to morphine milligram equivalents (MMEs). Secondary outcomes included extubation time and postoperative nausea and vomiting (PONV). **Results:** Median 24 h NRS was 3.0 (0–5.0) in the ESPB group and 2.5 (0–5.0) in the IPB + SAPB group. The between-group difference remained within the predefined two-point margin (95% CI: −0.8 to 1.2). Opioid consumption (*p* = 0.394), extubation time, and PONV incidence were comparable (all *p* > 0.05). No block-related complications occurred. **Conclusions:** IPB + SAPB was non-inferior to ESPB for postoperative analgesia in MICS. Despite requiring two injections, it remains an effective alternative. Larger trials are needed to confirm these findings.

## 1. Introduction

Minimally invasive techniques have significantly reduced the surgical trauma associated with traditional median sternotomy, enabling procedures such as aortic or mitral valve surgeries and coronary artery bypass grafting (CABG) to be performed through smaller incisions [1,2,3,4]. These approaches cause less disruption to chest wall structures and are linked to faster recovery, shorter hospital stays, and fewer complications, including infections and transfusion needs [1,2,3,5,6].

Although incisions are smaller, thoracotomy-based procedures may still cause significant postoperative pain, especially during early recovery [4,5,6,7,8,9,10]. This pain is worsened by breathing, coughing, and irritation of intercostal nerves due to rib spreading and chest tube insertion [6,7,8,10]. Inadequate pain control can prolong intensive care unit (ICU) stay, raise the risk of pulmonary complications and chronic postsurgical pain, and delay recovery [4,6,7].

Systemic opioids have traditionally been the cornerstone of analgesia in cardiac surgery. Although effective, their adverse effects—such as nausea, vomiting, urinary retention, and respiratory depression—can limit early mobilization [7]. To reduce opioid use, alternatives like thoracic epidural analgesia (TEA) and paravertebral blocks have been investigated. However, these techniques pose technical challenges and increased risks, especially in the anticoagulated patient population typical of minimally invasive cardiac surgery (MICS) [7,8,9,11].

Recently, the erector spinae plane block (ESPB) has emerged as a promising and safer regional anesthesia technique. It involves injecting local anesthetic between the erector spinae muscle and the transverse processes, providing multisegmental analgesia of the chest wall [10,12]. ESPB has been shown to reduce perioperative opioid use and support improved recovery outcomes [8,9,12].

Other fascial plane blocks, including the superficial serratus anterior plane block (SAPB), pectoserratus plane block (PSPB), and interpectoral plane block (IPB), have been investigated for their analgesic roles in MICS [8,9,13,14]. These techniques target the anterior and lateral chest wall and are often combined to provide broader sensory coverage [13,15,16,17,18,19].

In light of the increasing emphasis on multimodal and opioid-sparing strategies in cardiac anesthesia, this randomized controlled trial was designed as a prospective, double-blind, non-inferiority study to evaluate the effectiveness of two ultrasound-guided fascial plane block techniques. Specifically, we aimed to determine whether the combination of IPB and SAPB provides non-inferior postoperative analgesia compared to the widely used ESPB in adult patients undergoing MICS. The primary outcome was defined as the Numerical Rating Scale (NRS) pain score at 24 h postoperatively, with a pre-specified non-inferiority margin of two points. All interventions were applied after induction of general anesthesia and before the surgical incision to facilitate preemptive analgesia, improve technical success under optimal sonographic conditions, and minimize discomfort or logistical delays in the immediate postoperative period.

## 2. Materials and Methods

### 2.1. Study Design and Ethical Approval

This prospective, randomized, double-blind, non-inferiority clinical trial was conducted at Tekirdağ Namık Kemal University Hospital between 29 November 2022 and 15 November 2024. The study was approved by the Ethics Committee of Tekirdağ Namık Kemal University (Approval No: 2022.212.11.13; Date: 29 November 2022) and adhered to the Helsinki Declaration and CONSORT guidelines. It was registered at ClinicalTrials.gov (identifier: NCT05743231, registered on 24 February 2023). Written informed consent was obtained from all participants.

### 2.2. Study Population

Patients aged 18–75 years scheduled for their first MICS with American Society of Anesthesiologists (ASA) physical status I–III were eligible. Exclusion criteria included prior open-heart surgery, malignancy, abnormal coagulation, known allergy to local anesthetics, inability to operate a PCA device, or reoperation within 24 h postoperatively. This inclusion criterion was selected to minimize heterogeneity in pain perception and opioid sensitivity while maintaining external validity. Further studies with broader inclusion parameters and larger sample sizes are warranted. At our institution, the average operative duration is approximately 270–310 min for MIDCAB and 290–330 min for minimally invasive valve surgeries. These procedures constitute around 30% of all cardiac surgeries annually and are increasingly adopted as part of the institution’s enhanced recovery efforts.

### 2.3. Randomization and Blinding

Participants were randomized in a 1:1 ratio using a computer-generated list. Allocation was concealed in sealed opaque envelopes, revealed only before the block procedure. The anesthesiologist performing the blocks did not take part in postoperative assessments, to maintain blinding. Both patients and evaluators were blinded to group assignments.

### 2.4. Anesthesia Protocol

Standard intraoperative monitoring included noninvasive blood pressure, pulse oximetry, electrocardiography, invasive arterial pressure, and arterial blood gas analysis. Central venous access was established for hemodynamic monitoring. General anesthesia was induced with propofol (2 mg/kg), fentanyl (2 µg/kg), and rocuronium (0.6–0.8 mg/kg) and maintained with sevoflurane (1.0 MAC) and remifentanil infusion (0.1 µg/kg/min) in a 50% oxygen–air mixture. Remifentanil consumption was recorded and converted to morphine milligram equivalents (MMEs) using the standard equivalence of 1 mg remifentanil = 100 mg morphine, based on published opioid conversion ratios [20].

### 2.5. Regional Anesthesia Techniques

Blocks were performed after anesthesia induction and before surgical incision under sterile conditions using ultrasound guidance (Esaote MyLab Seven, Genova, Italy, linear probe 6–18 MHz). A single experienced anesthesiologist conducted all blocks.

**Figure 1:**
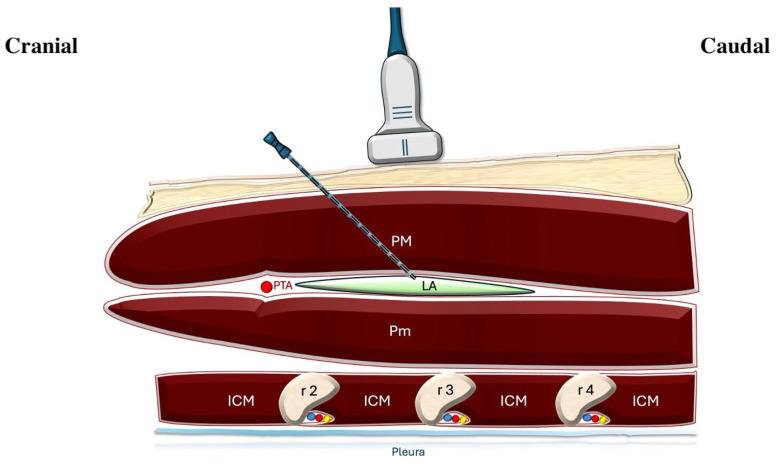
Illustration of ultrasound-guided interpectoral plane block. The ultrasound probe is placed over the pectoral region to visualize the pectoralis major and pectoralis minor muscles. A needle is advanced in-plane toward the interfascial plane between these muscles, where local anesthetic is deposited. The pectoral branch of the thoracoacromial artery serves as an anatomical landmark near the target region. The intercostal neurovascular bundles are located just inferior to each rib. PM: pectoralis major muscle; Pm: pectoralis minor muscle; LA: local anesthetic; PTA: pectoral branch of the thoracoacromial artery; ICM: intercostal muscle; r2: second rib; r3: third rib; r4: fourth rib.

In the IPB + SAPB group, a 22G, 50-mm Pajunk SonoPlex needle (Geisingen, Germany) was used for IPB between the pectoralis major and minor muscles with 15 mL 0.25% bupivacaine (Figure 1).

**Figure 2:**
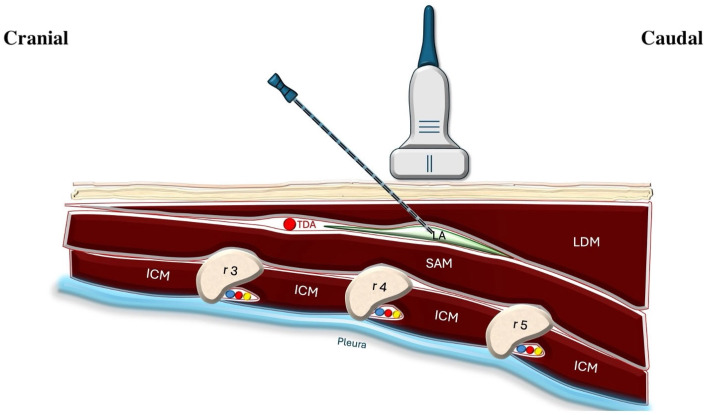
Illustration of the ultrasound-guided superficial serratus anterior plane block. The ultrasound probe is positioned to visualize the latissimus dorsi and serratus anterior muscle. A needle is inserted in-plane toward the fascial plane between these two muscles, and local anesthetic is administered. The thoracodorsal artery serves as a key anatomical landmark for correct placement. The intercostal neurovascular bundles are located just inferior to each rib. LDM: latissimus dorsi muscle; SAM: serratus anterior muscle; LA: local anesthetic; TDA: thoracodorsal artery; ICM: intercostal muscle; r3: third rib; r4: fourth rib; r5: fifth rib.

For the SAPB, 15 mL of 0.25% bupivacaine was injected between the serratus anterior and latissimus dorsi at the fifth rib level (Figure 2).

**Figure 3:**
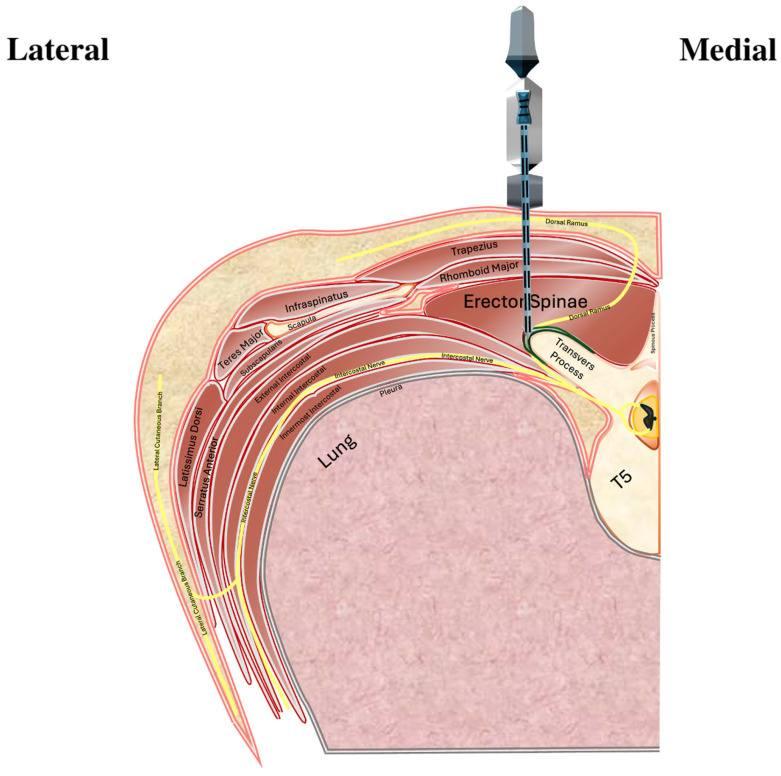
Illustration of ultrasound-guided erector spinae plane block. The ultrasound probe is placed longitudinally over the transverse process. A needle is advanced in-plane into the fascial plane between the erector spinae muscle and the transverse process, where local anesthetic is deposited. The drug spreads cranio-caudally, targeting both the dorsal and ventral rami of the spinal nerves. The pleura is visible beneath the injection site and serves as a critical structure to avoid.

In the ESPB group, a 22G, 80 mm Pajunk SonoPlex needle was used to administer 30 mL 0.25% bupivacaine between the erector spinae muscle and transverse process at T5 (Figure 3). Block laterality matched the surgical side.

### 2.6. Postoperative Management

In routine practice outside the study setting, postoperative analgesia in our institution for both minimally invasive and conventional cardiac surgeries includes IV paracetamol (1 g every 8 h), fentanyl PCA, and IV ketorolac (15 mg) for NRS > 4. Fascial plane blocks have been selectively used in minimally invasive procedures but are not standard in conventional approaches.

Following the surgery, patients were transferred to the cardiovascular ICU. Extubation criteria included spontaneous respiration, ABG stability, SpO_2_ > 92% on FiO_2_ < 40%, and hemodynamic stability. Pain was assessed using the Critical-Care Pain Observation Tool (CPOT) during intubation and the Numerical Rating Scale (NRS) at 6, 12, 24, and 48 h postoperatively, after patients were extubated.

Fentanyl PCA (10 µg/mL concentration; 25 µg bolus; 10 min lockout) was used. Total fentanyl consumption was recorded and converted to MMEs using the standard conversion factor of 100 µg fentanyl = 10 mg morphine, in accordance with the opioid conversion guidelines provided by the U.S. Centers for Disease Control and Prevention (CDC) [21]. Routine analgesia included IV paracetamol (1 g every 8 h). Rescue analgesia for NRS > 4 included IV ketorolac (15 mg) and, if needed, IV morphine (10 mg).

### 2.7. Outcome Measures

Primary outcome: NRS pain scores at 24 h postoperatively. Secondary outcomes: NRS pain scores at 6, 12, and 48 h; CPOT scores during intubation; 48 h total opioid consumption (MMEs); extubation time; and PONV incidence.

### 2.8. Sample Size Calculation

This study was designed as a non-inferiority trial, with the primary endpoint being the NRS pain score at 24 h postoperatively. The non-inferiority margin was pre-specified as two NRS points, based on both clinical judgment and prior literature. While the minimal clinically important difference (MCID) for acute postoperative pain is often cited as approximately one point, especially on the VAS scale [22], a two-point NRS difference has been considered an acceptable threshold for clinical relevance in other randomized studies evaluating regional anesthesia and multimodal analgesia interventions in acute surgical pain settings [23]. Furthermore, recent editorial guidance from the British Journal of Anaesthesia emphasizes that the non-inferiority margin should not be equated directly with the MCID, but rather determined through a combination of statistical reasoning and clinical acceptability, taking into account expected variability and the degree of efficacy loss that is deemed tolerable in clinical practice [24].

Based on this two-point margin, a standard deviation of 2, a power of 80%, a one-sided alpha of 0.05, and a total of 17 patients per group were required to demonstrate non-inferiority using a two-sample *t*-test. To account for potential dropouts and missing data, 20 patients per group (total *n* = 40) were enrolled. The sample size calculation was performed using G*Power v3.1 (Heinrich Heine University, Düsseldorf, Germany).

### 2.9. Statistical Analysis

Descriptive statistics were calculated as frequencies and percentages for categorical variables. The normality of continuous variables was assessed using the Shapiro–Wilk test. All continuous variables were presented as median [minimum–maximum]. Comparisons between block types were performed using the Mann–Whitney U test for continuous variables and Pearson’s Chi-square or Fisher’s exact test for categorical variables.

Missing data imputation was conducted using R programming language. The data distribution was evaluated using the Shapiro–Wilk test to determine the missing data mechanism, and imputation methods were selected based on missing data rates and variable distribution characteristics. For CPOT scores at 4 h and NRS scores at 6 h, which did not follow normal distribution, the K-nearest neighbor (KNN) method was employed. The knnImputation() function from the DMwR package was utilized to estimate each missing observation based on similar cases. For CPOT scores at 6 h, which showed closer adherence to normal distribution, mean imputation was performed using the base::mean() function. Had the missing data rate exceeded 30%, multiple imputation by chained equations (MICE) would have been recommended using the mice() function from the mice package to enhance model prediction power. The imputation methods were selected considering missing data mechanisms, variable distributions, and statistical consistency to maintain the reliability of analytical results.

Statistical analyses were conducted using a nonparametric longitudinal data analysis approach. The nparLD (Nonparametric Analysis of Longitudinal Data) method was chosen for evaluating repeated measurements, considering the ordinal nature of CPOT and NRS scores, violation of normality assumptions, and small sample size.

For repeated measurements analysis, the nparLD F1-LD-F1 model was implemented, and relative treatment effects (RTE) were calculated. Time effects, group effects, and time-group interactions were evaluated. Separate models were constructed for NRS and CPOT score longitudinal analyses. In subgroup analyses by surgery type (MIDCAB and valve surgery), between-group comparisons were performed using the Mann–Whitney U test and Fisher’s exact test.

To evaluate the non-inferiority hypothesis, the between-group difference in 24 h NRS scores was calculated, and the 95% confidence interval (CI) of this difference was compared against the pre-specified non-inferiority margin of two points. Non-inferiority was concluded if the entire CI fell within this margin. This analysis was performed using a one-sided 95% CI approach, consistent with standard recommendations for non-inferiority trials.

All analyses were performed using Jamovi (Version 2.3.28), JASP (Version 0.19.2), and R (version 4.4.2) software packages. Statistical significance was set at *p* ≤ 0.05.

## 3. Results

**Figure 4:**
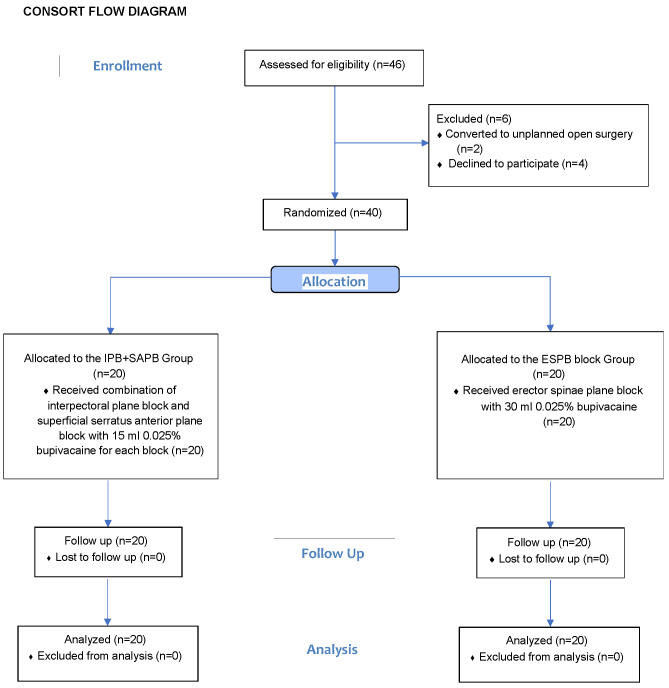
CONSORT flow diagram of participant enrollment. The chart outlines the study process, including the number of patients assessed for eligibility, randomized, allocated to either the ESPB or IPB + SAPB group, followed up, and included in the final analysis. Reasons for exclusion or loss to follow up are provided at each stage, in accordance with CONSORT guidelines. ESPB: erector spinae plane block; IPB: interpectoral plane block; SAPB: superficial serratus anterior plane block.

A total of 46 patients were screened for eligibility, of whom six were excluded (two due to conversion to open surgery and four who declined participation). The remaining 40 patients were randomized equally to receive either ESPB (*n* = 20) or IPB + SAPB (*n* = 20) (Figure 4).

Baseline demographic data, ASA physical status distribution, type of surgery, and operative/anesthesia durations were similar between the ESPB and IPB + SAPB groups (Table 1).

**Figure 5:**
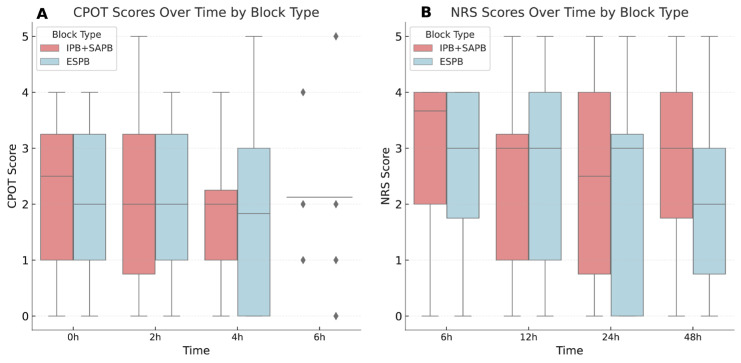
Changes in pain scores over time following minimally invasive cardiac surgery with ESPB and IPB + SAPB. (**A**) Box plots representing CPOT scores at 0, 2, 4, and 6 h postoperatively. A significant time effect was observed (*p* = 0.029), but no group or time × group interaction effect was found. Outlier values at the 6-h time point are represented by diamond symbols. (**B**) Box plots representing NRS scores at 6, 12, 24, and 48 h postoperatively, recorded during the postoperative period when patients were awake and extubated. No statistically significant differences were detected between the groups. ESPB: erector spinae plane block; IPB: interpectoral plane block; SAPB: superficial serratus anterior plane block; CPOT: critical-care pain observation tool; NRS: numeric rating scale.

Pain scores and treatment effects were comparable between groups, with no significant differences in remifentanil consumption, CPOT scores, or NRS scores (Table 2, Figure 5). The analysis confirmed non-inferiority of IPB + SAPB compared to ESPB for postoperative analgesia. The median NRS score at 24 h was 3.0 (0–5.0) in the ESPB group and 2.5 (0–5.0) in the IPB + SAPB group. The between-group difference was −0.5 points, with a 95% confidence interval of −0.8 to 1.2. Since the entire confidence interval was within the pre-specified non-inferiority margin of two points, non-inferiority was confirmed.

Postoperative analgesic requirements and recovery parameters were comparable between groups (Table 3). Both ESPB and IPB + SAPB provided similar cumulative opioid-sparing effects without significant differences in rescue analgesic use. Recovery outcomes, including extubation time and PONV rates, were also similar, supporting the non-inferiority of IPB + SAPB in the multimodal analgesia protocol.

Subgroup analysis revealed no significant differences in total opioid consumption, extubation time, PONV incidence, or 24 h NRS scores between ESPB and IPB + SAPB, in either the MIDCAB or valve surgery groups (Table 4). In the valve subgroup, findings should be interpreted cautiously due to the small ESPB sample size (*n* = 4).

## 4. Discussion

This randomized, controlled, single-center non-inferiority trial compared the analgesic efficacy of IPB + SAPB with ESPB in MICS. The primary objective was to assess whether IPB + SAPB is non-inferior to ESPB in postoperative pain control and opioid reduction.

Both techniques effectively managed postoperative pain, showing no significant differences in pain scores, opioid consumption, time to extubation, or PONV. These findings support the non-inferiority of IPB + SAPB and suggest it may be a valid alternative to ESPB in multimodal analgesia protocols for MICS.

Although ESPB is widely used for its broad thoracic dermatomal coverage, IPB + SAPB provides more focused analgesia to the anterior and lateral chest wall—making it potentially more suitable for anterior thoracic incisions. Therefore, block selection should consider surgical approach, anatomy, patient comorbidities, and clinician experience.

The ESPB has been extensively studied in thoracic and cardiac surgeries for its multisegmental analgesia via craniocaudal diffusion along the fascial plane near the erector spinae muscle [25,26]. Xin et al. showed that single-shot ESPB reduces postoperative NRS scores in MIDCAB [8]. In our study, ESPB served as the reference and provided comparable analgesic efficacy to the IPB + SAPB combination.

These findings confirm ESPB as a reliable option for postoperative analgesia in MICS. While some studies have questioned its anterior chest wall coverage due to variable anterior spread [8,27,28], no such limitations were observed in our patients. Despite reports like Hoogma et al. showing no significant benefit in minimally invasive mitral valve surgery [10], our data support ESPB as a safe, effective, and reproducible technique in enhanced recovery protocols. Differences in findings may be attributed to methodological factors such as control group selection, surgical approach variability, and block performance techniques.

Unlike ESPB, which was the reference in our study, the IPB targets the anterior chest wall and is well-suited for MICS procedures involving anterior thoracotomy or parasternal incisions. By anesthetizing the medial and lateral pectoral nerves, IPB reduces pain from sternal retraction and muscle trauma [29]. Although widely studied in breast and thoracic surgery [30], its role in cardiac surgery remains underexplored.

Vinzant et al. compared the pectoserratus plane block (PSPB) to paravertebral blocks in minimally invasive mitral valve surgery, showing comparable opioid-sparing effects [3]. In our study, combining IPB with SAPB aimed to provide broader anterior and lateral chest wall coverage. This may be particularly useful in surgeries where anterior thoracic innervation predominates and focused analgesia is essential.

The SAPB has been widely used in thoracic surgery, rib fractures, and MICS for targeting the lateral branches of intercostal nerves within the fascial plane between the serratus anterior and latissimus dorsi muscles [5,31,32]. Gautam et al. and Saikat et al. showed its opioid-sparing and mobilization benefits [6,19]. In this study, SAPB was combined with IPB to provide broader thoracic wall analgesia compared to ESPB.

This combination aimed to enhance lateral chest wall analgesia as part of a comprehensive multimodal strategy. Although SAPB yields favorable early outcomes, its single-shot duration is limited. To address this, continuous SAPB using catheter-based infusion has been proposed to prolong and stabilize postoperative analgesia [18,33].

This study found no significant differences between ESPB and the IPB + SAPB regarding postoperative pain, opioid use, or recovery, supporting the non-inferiority of IPB + SAPB in MICS. Despite requiring two injections, IPB + SAPB presents several practical advantages that may justify its use in selected cases. Unlike ESPB, which often necessitates lateral decubitus or sitting positioning under general anesthesia for optimal ultrasound visualization, both IPB and SAPB can be performed in the supine position—reducing procedural complexity and the need for patient repositioning. This is particularly advantageous in cardiac surgery, where maintaining patient stability and minimizing line displacement are critical. In clinical contexts where anterior-lateral chest wall coverage is prioritized or where posterior access is limited, IPB + SAPB may therefore serve as a logistically efficient and anatomically suitable alternative to ESPB. For procedures involving lateral or posterolateral thoracic incisions, however, ESPB may still be preferable due to its extensive multisegmental spread.

Although no block failures were encountered in our cohort, technical success may vary depending on sonographic visualization, anatomical variation, and operator skill. ESPB may also be more technically demanding in certain setups due to the need for lateral or prone positioning, while IPB and SAPB offer procedural simplicity by allowing supine performance, which is particularly advantageous in cardiac surgical settings. In our experience, both IPB and SAPB were reliably performed in the supine position without complications, contributing to procedural feasibility in the cardiac surgical population.

Additionally, both ESPB and IPB + SAPB were performed as single-shot techniques in this study. Although effective in the immediate postoperative period, their duration may be limited compared to catheter-based continuous infusions. This could impact sustained pain control beyond the initial 24–48 h. Future trials should explore the feasibility and analgesic benefits of continuous infusion strategies, which may prolong efficacy, reduce variability in block duration, and enhance recovery trajectories.

Technique selection may depend on surgical site, anatomy, and clinician preference. ESPB may be preferable for lateral or posterolateral incisions due to its multisegmental dermatomal spread, while IPB + SAPB may better target anterior thoracic regions. Both techniques offer safe, less invasive alternatives to neuraxial blocks. ESPB provides extended coverage, whereas SAPB might benefit from catheter-based infusion to prolong its effect.

This study has several limitations. Long-term outcomes, such as chronic post-surgical pain and functional recovery, were not assessed. The single-center design may limit generalizability, and larger multicenter trials are needed to confirm these findings. The subgroup comparison for valve surgery patients should be interpreted with caution due to small sample size, particularly in the ESPB group (*n* = 4). These analyses are exploratory and not powered to detect definitive differences. Further studies with catheter-based continuous techniques are needed to validate our findings and enhance postoperative analgesic consistency.

Although a formal cost-effectiveness analysis was not performed, both ESPB and IPB + SAPB rely on single-shot techniques with minimal additional material requirements. IPB + SAPB may increase procedural time due to dual injections but may reduce patient repositioning needs by allowing supine administration. Future studies should incorporate economic evaluations to better inform technique selection in resource-limited or high-volume surgical settings.

## 5. Conclusions

This randomized non-inferiority trial demonstrates that the combination of IPB + SAPB provides postoperative analgesia comparable to the ESPB in patients undergoing MICS. With similar pain scores, opioid consumption, and recovery outcomes, IPB + SAPB is a viable alternative within multimodal analgesia protocols. While ESPB offers broad dermatomal coverage, IPB + SAPB may provide more targeted anterior-lateral analgesia, especially for anterior thoracic incisions. Both techniques were safe and opioid-sparing. Future studies should explore catheter-based continuous infusion and assess long-term outcomes. These findings support the growing role of anterior-lateral fascial plane blocks as effective alternatives to ESPB in MICS.

## Figures and Tables

**Table 1:** Baseline demographic and clinical characteristics of patients undergoing minimally invasive cardiac surgery with different regional anesthesia techniques.

	ESPB (*n* = 20)	IPB + SAPB (*n* = 20)	*p*
Age	64.5 (50.0–74.0)	62.0 (51.0–73.0)	*0.507*
Gender (M/F)	13/7	14/6	*0.999*
BMI (kg/m^2^)	23.6 (19.0–29.5)	23.1 (18.7–29.6)	*0.968*
EuroSCORE	3.5 (1.6–7.3)	3.2 (1.6–5.7)	*0.655*
ASA (II/III)	8/12	10/10	*0.751*
Surgery type (MIDCAB/minimally invasive valve)	16/4	10/10	*0.097*
Surgery length (min)	300.0 (252.0–336.0)	294.0 (246.0–324.0)	*0.978*
Anesthesia length (min)	357.0 (319.0–403.0)	356.5 (315.0–390.0)	*0.882*

Data are presented as median (minimum–maximum) or number where appropriate. BMI values were calculated as weight in kilograms divided by height in meters squared. EuroSCORE indicates the predicted operative mortality. *p* values are italicized; *p* ≤ 0.05 was considered statistically significant. ESPB: erector spinae plane block; IPB: interpectoral plane block; SAPB: superficial serratus anterior plane block; M: male; F: female; BMI: body mass index; ASA: American Society of Anesthesiologists; MIDCAB: minimally invasive direct coronary artery bypass; min: minutes; kg/m^2^: kilograms per square meter.

**Table 2:** Longitudinal assessment of pain scores and treatment effects between groups.

	ESPB (*n* = 20)	IPB + SAPB (*n* = 20)	*p*
Intraoperative Parameters			
Remifentanil (μg)	2468.0 (1803.0–3018.0)	2382.0 (1814.0–3199.0)	*0.925*
Remifentanil MMEs (mg)	6.2 (4.5–7.6)	6.0 (4.5–8.0)	*0.925*
			RTE
CPOT Scores †			
Baseline	2.0 (0.0–4.0)	2.5 (0.0–4.0)	0.512/0.543
2 h	2.0 (0.0–4.0)	2.0 (0.0–5.0)	0.495/0.491
4 h	2.0 (0.0–5.0)	2.0 (0.0–4.0)	0.425/0.424
6 h	1.5 (0.0–5.0)	2.0 (1.0–4.0)	0.549/0.560
CPOT Analysis			
Group Effect	*p = 0.900*		
Time Effect	*p = 0.029*		
Group × Time Interaction	*p = 0.933*		
NRS Scores ‡			
6 h	3.0 (0.0–4.0)	4.0 (0.0–4.0)	0.510/0.562
12 h	3.0 (0.0–5.0)	3.0 (0.0–5.0)	0.505/0.512
24 h	3.0 (0.0–5.0)	2.5 (0.0–5.0)	0.475/0.497
48 h	2.0 (0.0–5.0)	3.0 (0.0–5.0)	0.405/0.534
Longitudinal Analysis Results			
NRS Analysis			
Group Effect	*p = 0.517*		
Time Effect	*p = 0.201*		
Group × Time Interaction	*p = 0.238*		

Data are presented as median (minimum–maximum). RTE values are presented as ESPB/IPB + SAPB. Convert the sentence to: *p* values are italicized; *p* ≤ 0.05 was considered statistically significant. † CPOT scores were assessed during the early postoperative period. ‡ NRS scores were evaluated over an extended 48 h period. Nonparametric longitudinal data analysis (nparLD) was performed for repeated measurements. The analysis revealed a significant time effect for CPOT scores (*p* = 0.029), while no significant group or interaction effects were observed for either CPOT or NRS measurements. RTE values represent the relative magnitude of treatment effects between groups at each time point. CPOT: critical-care pain observation tool; NRS: numeric rating scale; ESPB: erector spinae plane block; IPB: interpectoral plane block; SAPB: superficial serratus anterior plane block; MMEs: morphine milligram equivalents; RTE: relative treatment effect; μg: micrograms; mg: milligrams.

**Table 3:** Comparison of postoperative analgesic use and recovery outcomes.

	ESPB (*n* = 20)	IPB + SAPB (*n* = 20)	*p*
Analgesic Requirements			
PCA Fentanyl (μg)	1725.0 (900.0–2575.0) [IQR: 1331.2–2225.0]	2025.0 (975.0–2625.0) [IQR: 1650.0–2293.7]	*0.432*
PCA MMEs (mg)	172.5 (90.0–257.5) [IQR: 133.1–222.5]	202.5 (97.5–262.5) [IQR: 165.0–229.4]	*0.432*
Rescue Analgesic Count	3 (15.0)	6 (30.0)	*0.451*
Total MME (mg)	178.0 (108.0–265.0) [IQR: 138.5–230.8]	208.5 (103.0–267.0) [IQR: 170.8–235.5]	*0.394*
Recovery Parameters			
Extubation Time (h)	5.3 (3.1–7.1)	5.0 (3.0–7.3)	*0.675*
ICU CO_2_ Level	41.9 (29.5–48.9)	39.5 (31.1–49.6)	*0.925*
Drainage Amount (mL)	250.0 (150.0–300.0)	200.0 (150.0–250.0)	*0.145*
NIRS Change	−4.6 (−7.7–0.9)	−5.1 (−9.9–2.0)	*0.218*
PONV, *n* (%)	2 (10.0)	4 (20.0)	*0.661*

Data are presented as median (minimum–maximum) or number (%) where appropriate. PCA consumption is reported for the first 48 postoperative hours. Total MME includes both PCA consumption and rescue analgesics. IQR (interquartile range) values are provided in square brackets to illustrate data dispersion between the 25th and 75th percentiles. *p* values are italicized; *p* ≤ 0.05 was considered statistically significant. ESPB: erector spinae plane block; IPB: interpectoral plane block; SAPB: superficial serratus anterior plane block; PCA: patient-controlled analgesia; MMEs: morphine milligram equivalents; ICU: intensive care unit; NIRS: near-infrared spectroscopy; PONV: postoperative nausea and vomiting; μg: micrograms; mg: milligrams; h: hours; mL: milliliters.

**Table 4:** Subgroup analysis of postoperative outcomes by surgery type.

	MIDCAB			Valve Surgery		
	ESPB (*n* = 16)	IPB + SAPB (*n* = 10)	*p*	ESPB (*n* = 4)	IPB + SAPB (*n* = 10)	*p*
Total MMEs (mg)	178.0 (108.0–265.0)	209.0 (103.0–263.0)	*0.916*	162.0 (124.0–246.0)	208.0 (140.0–267.0)	*0.179*
Extubation Time (h)	5.2 (3.1–7.1)	5.5 (3.7–7.3)	*0.341*	5.4 (4.9–5.9)	4.8 (3.0–6.5)	*0.076*
PONV *n* (%)	2 (12.5)	3 (30.0)	*0.340*	0 (0.0)	1 (10.0)	*0.999*
NRS at 24 h	3.0 (0.0–5.0)	4.0 (0.0–4.0)	*0.345*	2.0 (0.0–4.0)	2.0 (0.0–5.0)	*0.827*

Data are presented as median (minimum–maximum) or number (%) where appropriate. Total MMEs represent the cumulative opioid consumption during the first 48 postoperative hours. NRS was assessed using a 0–10 scale. *p* values are italicized; *p* ≤ 0.05 was considered statistically significant. ESPB: erector spinae plane block; IPB: interpectoral plane block; SAPB: superficial serratus anterior plane block; MIDCAB: minimally invasive direct coronary artery bypass; MME: morphine milligram equivalents; PONV: postoperative nausea and vomiting; NRS: numeric rating scale; mg: milligrams.

## Data Availability

The datasets generated and/or analyzed during the current study are not publicly available: but are available from the corresponding author on reasonable request.

## Abbreviations

The following abbreviations are used in this manuscript:

ABG	Arterial blood gas
ASA	American Society of Anesthesiologists
BMI	Body mass index
CONSORT	Consolidated Standards of Reporting Trials
CPOT	Critical-Care Pain Observation Tool
ESPB	Erector spinae plane block
FiO_2_	Fraction of inspired oxygen
h	Hours
ICM	Intercostal muscle
ICU	Intensive care unit
IPB	Interpectoral plane block
IV	Intravenous
kg/m^2^	Kilograms per square meter
LA	Local anesthetic
LDM	Latissimus dorsi muscle
MAC	Minimum alveolar concentration
MICS	Minimally invasive cardiac surgery
MIDCAB	Minimally invasive direct coronary artery bypass
mg	Milligrams
min	Minutes
mL	Milliliters
MMEs	Morphine milligram equivalents
NIRS	Near-infrared spectroscopy
NRS	Numeric rating scale
PCA	Patient-controlled analgesia
PM	Pectoralis major muscle
Pm	Pectoralis minor muscle
PONV	Postoperative nausea and vomiting
PTA	Pectoral branch of the thoracoacromial artery
RTE	Relative treatment effect
SAM	Serratus anterior muscle
SAPB	Superficial serratus anterior plane block
TDA	Thoracodorsal artery
µg	Micrograms
